# 2-Amino­anilinium 4-methyl­benzene­sulfonate

**DOI:** 10.1107/S2414314620002308

**Published:** 2020-02-21

**Authors:** Kedar U. Narvekar, Bikshandarkoil R. Srinivasan

**Affiliations:** a School of Chemical Sciences, Goa University PO, Goa 403206, India; University of Aberdeen, Scotland

**Keywords:** crystal structure, benzene-1,2-di­amine, N—H⋯O hydrogen bonds

## Abstract

In the extended structure of the title mol­ecular salt, C_6_H_9_N_2_^+^·C_7_H_7_O_3_S^−^, the cations and anions are linked by N—H⋯O hydrogen bonds to generate [010] chains.

## Abstract 2

In the extended structure of the title mol­ecular salt, the cations and anions are linked by N—H⋯O hydrogen bonds to generate [010] chains.

## Graphical abstract

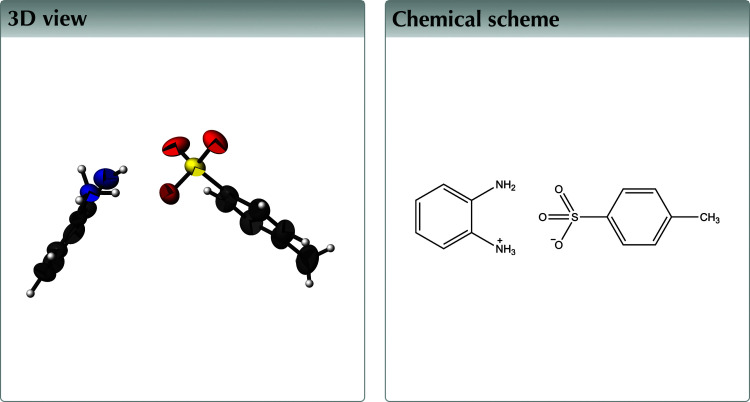

## Structure description

As part of an ongoing research programme, we are investigating the structural chemistry of the mol­ecular salts of benzene-1,2-di­amine. In a recent data report we described the crystal structure of benzene-1,2-diaminium bis­(4-methyl­benzene-1-sulfonate), **2** (Narvekar & Srinivasan, 2020 ▸). The structure of another mol­ecular salt of 4-methyl­benzene-1-sulfonic acid containing both mono and diprotonated cations of benzene-1,2-di­amine, namely 2-amino­anilinium benzene-1,2-diaminium tris­(4-methyl­benzene-1-sulfonate), **3**, was reported earlier (Amirthakumar *et al.*, 2018 ▸). The Cambridge Structural Database (CSD; Groom *et al.*, 2016 ▸) lists several structurally characterized salts of benzene-1,2-di­amine containing the monoprotonated 2-amino­anilinium cation or the diprotonated benzene-1,2-diaminium dication.

In this report, we describe the crystal structure of the title salt, **1**, which is the third anhydrous compound that can be isolated from the benzene-1,2-di­amine/4-methyl­benzene-1-sulfonic acid/water system. The title salt was obtained by an aqueous reaction of the aromatic di­amine with 4-methyl­benzene-1-sulfonic acid in a 2:1 molar ratio, unlike **2**, which was crystallized from a 1:2 reaction.

**Figure 1:**
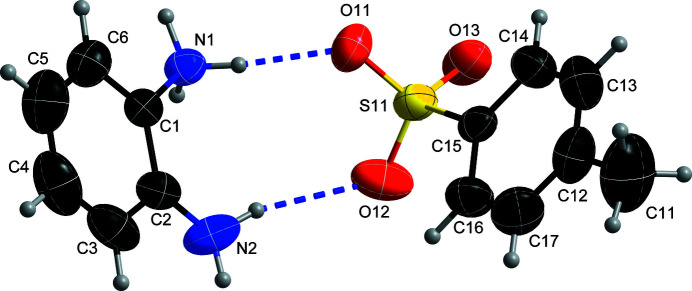
The mol­ecular structure of **1** with displacement ellipsoids drawn at 50% probability level. Hydrogen bonds are shown as blue dashed lines.

The asymmetric unit of **1** consists of one 2-amino­anilinium cation and one 4-methyl­benzene-1-sulfonate anion (Fig. 1 ▸) with all atoms located in general positions. The geometric parameters of the cation and the anion are in their normal ranges and are in agreement with previously reported data (Mishra & Pallepogu, 2018 ▸; Narvekar & Srinivasan, 2020 ▸).

**Figure 2:**
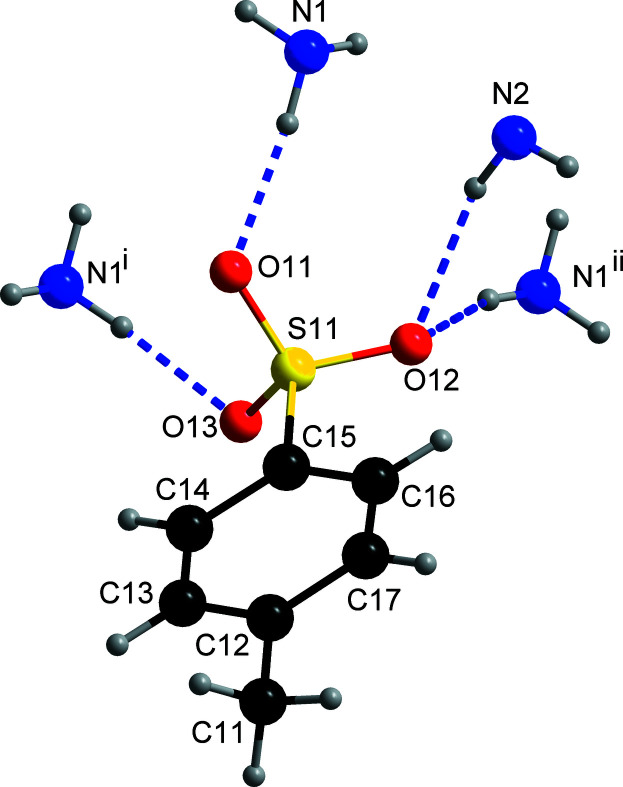
The hydrogen-bonding scheme around the 4-methyl­benzene-1-sulfonate anion. Symmetry codes: (i) *-x* + 1, −*y*, −*z* + 1; (ii) −*x* + 1, −*y* + 1, −*z* + 1.

**Figure 3:**
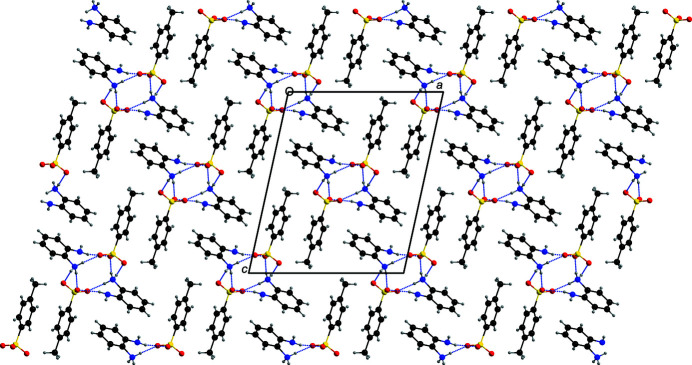
A view down [010] of the packing in **1** with N—H⋯O hydrogen bonds shown as dashed lines.

All the oxygen atoms attached to the sulfur atom of the sulfonate moiety of the anion function as hydrogen-bond acceptors (Fig. 2 ▸), while four of the five H atoms attached to the N atoms of the 2-amino­anilinium cation function as hydrogen-bond donors, resulting in a total of four N—H⋯O hydrogen bonds (with two occurring within the arbitrarily chosen asymmetric unit; Table 1 ▸). Thus, the cations and the anions are linked only *via* N—H⋯O hydrogen bonds, as observed earlier for **2**. The extended structure of **1** features [010] hydrogen-bonded chains, with adjacent cations and anions related by the 2_1_ screw axis (Fig. 3 ▸).

## Synthesis and crystallization

Freshly recrystallized benzene-1,2-di­amine (216 mg, 1 mmol) was dissolved in aqueous ethanol (25–30 ml). Into this, an aqueous solution of 4-methyl­benzene-1-sulfonic acid (190 mg, 1 mmol) was added. The clear reaction mixture thus obtained was left aside for crystallization. After a few days crystals of **1** in the form of colourless blocks slowly separated. The crystals were filtered and dried in air. Yield 45%.

## Refinement

Crystal data, data collection and structure refinement details are summarized in Table 2 ▸.

## Supplementary Material

Crystal structure: contains datablock(s) I, global. DOI: 10.1107/S2414314620002308/hb4337sup1.cif

Structure factors: contains datablock(s) I. DOI: 10.1107/S2414314620002308/hb4337Isup2.hkl

Supporting information file. DOI: 10.1107/S2414314620002308/hb4337Isup3.cml

CCDC reference: 1985013

Additional supporting information:  crystallographic information; 3D view; checkCIF report

## Figures and Tables

**Table 1:** Hydrogen-bond geometry (Å, °)

*D*—H⋯*A*	*D*—H	H⋯*A*	*D*⋯*A*	*D*—H⋯*A*
N1—H1*A*⋯O11	0.89	1.89	2.7767 (17)	174
N1—H1*B*⋯O13^i^	0.89	1.93	2.8111 (17)	171
N1—H1*C*⋯O12^ii^	0.89	1.92	2.7914 (17)	165
N2—H2*A*⋯O12	0.90 (2)	2.22 (2)	3.097 (3)	165 (2)

Symmetry codes: (i) 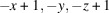 ; (ii) 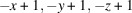 .

**Table 2:** Experimental details

Crystal data	
Chemical formula	C_6_H_9_N_2_^+^·C_7_H_7_O_3_S^−^
*M*_r_	280.34
Crystal system, space group	Monoclinic, *P*2_1_/*n*
Temperature (K)	293
*a*, *b*, *c* (Å)	14.6392 (5), 5.7111 (2), 17.5295 (6)
β (°)	102.349 (1)
*V* (Å^3^)	1431.66 (9)
*Z*	4
Radiation type	Mo *K*α
μ (mm^−1^)	0.23
Crystal size (mm)	0.45 × 0.37 × 0.33

Data collection	
Diffractometer	Bruker D8 Quest ECO
Absorption correction	Multi-scan (*SADABS*; Krause *et al.*, 2015 ▸)
*T*_min_, *T*_max_	0.682, 0.746
No. of measured, independent and observed [*I* > 2σ(*I*)] reflections	22028, 4362, 3253
*R*_int_	0.027
(sin θ/λ)_max_ (Å^−1^)	0.715

Refinement	
*R*[*F* ^2^ > 2σ(*F* ^2^)], *wR*(*F* ^2^), *S*	0.042, 0.127, 1.06
No. of reflections	4362
No. of parameters	182
H-atom treatment	H atoms treated by a mixture of independent and constrained refinement
Δρ_max_, Δρ_min_ (e Å^−3^)	0.21, −0.31

Computer programs: *APEX3* and *SAINT* (Bruker, 2019 ▸), *SHELXT2014* (Sheldrick, 2008 ▸), *SHELXL2018* (Sheldrick, 2015 ▸), *OLEX2* (Dolomanov *et al.*, 2009 ▸) and *shelXle* (Hübschle *et al.*, 2011 ▸).

## full crystallographic data

### Crystal data

**Table:**

C_6_H_9_N_2_^+^·C_7_H_7_O_3_S^−^	*F*(000) = 592
*M**_r_* = 280.34	*D*_x_ = 1.301 Mg m^−^^3^
Monoclinic, *P*2_1_/*n*	Mo *K*α radiation, λ = 0.71073 Å
*a* = 14.6392 (5) Å	Cell parameters from 7623 reflections
*b* = 5.7111 (2) Å	θ = 2.9–30.2°
*c* = 17.5295 (6) Å	µ = 0.23 mm^−^^1^
β = 102.349 (1)°	*T* = 293 K
*V* = 1431.66 (9) Å^3^	Block, colourless
*Z* = 4	0.45 × 0.37 × 0.33 mm

### Data collection

**Table:**

Bruker D8 Quest ECO diffractometer	3253 reflections with *I* > 2σ(*I*)
Radiation source: Sealed Tube	*R*_int_ = 0.027
φ and ω scans	θ_max_ = 30.5°, θ_min_ = 2.9°
Absorption correction: multi-scan (SADABS; Krause *et al.*, 2015)	*h* = −20→20
*T*_min_ = 0.682, *T*_max_ = 0.746	*k* = −8→8
22028 measured reflections	*l* = −25→25
4362 independent reflections	

### Refinement

**Table:**

Refinement on *F*^2^	Primary atom site location: dual
Least-squares matrix: full	Hydrogen site location: mixed
*R*[*F*^2^ > 2σ(*F*^2^)] = 0.042	H atoms treated by a mixture of independent and constrained refinement
*wR*(*F*^2^) = 0.127	*w* = 1/[σ^2^(*F*_o_^2^) + (0.0529*P*)^2^ + 0.3765*P*] where *P* = (*F*_o_^2^ + 2*F*_c_^2^)/3
*S* = 1.06	(Δ/σ)_max_ = 0.010
4362 reflections	Δρ_max_ = 0.21 e Å^−^^3^
182 parameters	Δρ_min_ = −0.31 e Å^−^^3^
0 restraints	

### Special details

**Table:**

Geometry. All esds (except the esd in the dihedral angle between two l.s. planes) are estimated using the full covariance matrix. The cell esds are taken into account individually in the estimation of esds in distances, angles and torsion angles; correlations between esds in cell parameters are only used when they are defined by crystal symmetry. An approximate (isotropic) treatment of cell esds is used for estimating esds involving l.s. planes.

### Fractional atomic coordinates and isotropic or equivalent isotropic displacement parameters (Å^2^)

**Table:**

	*x*	*y*	*z*	*U*_iso_*/*U*_eq_	
N1	0.36804 (8)	0.2361 (2)	0.46460 (7)	0.0440 (3)	
H1A	0.414876	0.196763	0.442076	0.066*	
H1B	0.350598	0.111606	0.488514	0.066*	
H1C	0.386780	0.349023	0.499410	0.066*	
N2	0.37965 (15)	0.6564 (3)	0.38220 (11)	0.0700 (5)	
H2B	0.3826 (18)	0.763 (5)	0.3528 (16)	0.095 (9)*	
H2A	0.4355 (16)	0.584 (4)	0.3963 (13)	0.076 (7)*	
C1	0.28909 (9)	0.3195 (2)	0.40520 (8)	0.0394 (3)	
C2	0.29793 (11)	0.5272 (3)	0.36608 (8)	0.0468 (3)	
C3	0.21843 (15)	0.6058 (3)	0.31307 (10)	0.0657 (5)	
H3	0.221090	0.744552	0.285849	0.079*	
C4	0.13647 (15)	0.4805 (5)	0.30077 (12)	0.0804 (7)	
H4	0.084179	0.536909	0.265797	0.096*	
C5	0.13039 (14)	0.2741 (5)	0.33902 (13)	0.0774 (6)	
H5	0.074746	0.189818	0.329632	0.093*	
C6	0.20756 (11)	0.1926 (3)	0.39160 (10)	0.0552 (4)	
H6	0.204453	0.052101	0.417768	0.066*	
S11	0.59680 (3)	0.22022 (7)	0.39473 (2)	0.04691 (12)	
O11	0.51524 (8)	0.0815 (2)	0.40070 (7)	0.0599 (3)	
O12	0.58218 (10)	0.4676 (2)	0.40655 (7)	0.0702 (4)	
O13	0.68108 (8)	0.1290 (2)	0.44434 (7)	0.0647 (3)	
C11	0.6391 (2)	0.0983 (7)	0.06127 (15)	0.1203 (11)	
H11A	0.607486	−0.041356	0.039393	0.180*	
H11B	0.704391	0.086390	0.060695	0.180*	
H11C	0.612753	0.231288	0.030895	0.180*	
C12	0.62771 (14)	0.1275 (4)	0.14452 (12)	0.0720 (5)	
C13	0.65754 (14)	−0.0419 (4)	0.19988 (13)	0.0732 (5)	
H13	0.684183	−0.178430	0.185581	0.088*	
C14	0.64892 (12)	−0.0142 (3)	0.27597 (11)	0.0609 (4)	
H14	0.669112	−0.131566	0.312468	0.073*	
C15	0.61010 (10)	0.1889 (3)	0.29789 (9)	0.0444 (3)	
C16	0.58045 (14)	0.3615 (3)	0.24378 (11)	0.0619 (4)	
H16	0.554920	0.499363	0.258252	0.074*	
C17	0.58897 (16)	0.3279 (4)	0.16747 (12)	0.0764 (6)	
H17	0.567940	0.443925	0.130691	0.092*	

### Atomic displacement parameters (Å^2^)

**Table:**

	*U*^11^	*U*^22^	*U*^33^	*U*^12^	*U*^13^	*U*^23^
N1	0.0443 (6)	0.0444 (6)	0.0411 (6)	−0.0028 (5)	0.0042 (5)	0.0072 (5)
N2	0.0852 (13)	0.0577 (10)	0.0664 (10)	−0.0219 (9)	0.0146 (9)	0.0133 (8)
C1	0.0403 (6)	0.0410 (7)	0.0348 (6)	−0.0001 (5)	0.0037 (5)	−0.0034 (5)
C2	0.0600 (9)	0.0424 (7)	0.0368 (7)	0.0031 (6)	0.0077 (6)	−0.0008 (6)
C3	0.0862 (13)	0.0619 (11)	0.0450 (8)	0.0282 (10)	0.0050 (8)	0.0040 (8)
C4	0.0615 (11)	0.1129 (19)	0.0574 (11)	0.0317 (12)	−0.0082 (8)	−0.0091 (12)
C5	0.0463 (9)	0.1129 (18)	0.0668 (12)	−0.0095 (10)	−0.0019 (8)	−0.0164 (12)
C6	0.0523 (9)	0.0601 (10)	0.0518 (8)	−0.0128 (7)	0.0083 (7)	−0.0102 (7)
S11	0.0464 (2)	0.0440 (2)	0.0472 (2)	−0.00369 (15)	0.00294 (14)	0.00788 (15)
O11	0.0509 (6)	0.0685 (8)	0.0641 (7)	−0.0118 (6)	0.0208 (5)	−0.0027 (6)
O12	0.1021 (10)	0.0465 (7)	0.0531 (7)	0.0017 (7)	−0.0033 (6)	−0.0028 (5)
O13	0.0512 (6)	0.0777 (8)	0.0597 (7)	−0.0014 (6)	−0.0007 (5)	0.0279 (6)
C11	0.117 (2)	0.184 (3)	0.0711 (15)	0.009 (2)	0.0443 (15)	−0.0057 (19)
C12	0.0600 (10)	0.0984 (16)	0.0623 (11)	−0.0045 (11)	0.0240 (9)	−0.0010 (11)
C13	0.0648 (11)	0.0780 (13)	0.0803 (13)	0.0103 (10)	0.0235 (10)	−0.0079 (11)
C14	0.0588 (10)	0.0544 (10)	0.0684 (11)	0.0089 (8)	0.0111 (8)	0.0072 (8)
C15	0.0361 (6)	0.0440 (7)	0.0513 (8)	−0.0042 (5)	0.0057 (5)	0.0086 (6)
C16	0.0720 (11)	0.0550 (9)	0.0590 (10)	0.0110 (8)	0.0146 (8)	0.0159 (8)
C17	0.0873 (14)	0.0843 (15)	0.0586 (11)	0.0096 (12)	0.0179 (10)	0.0261 (10)

### Geometric parameters (Å, º)

**Table:**

N1—C1	1.4601 (17)	S11—O12	1.4506 (13)
N1—H1A	0.8900	S11—O11	1.4554 (12)
N1—H1B	0.8900	S11—C15	1.7586 (16)
N1—H1C	0.8900	C11—C12	1.513 (3)
N2—C2	1.382 (2)	C11—H11A	0.9600
N2—H2B	0.81 (3)	C11—H11B	0.9600
N2—H2A	0.90 (2)	C11—H11C	0.9600
C1—C6	1.373 (2)	C12—C13	1.374 (3)
C1—C2	1.390 (2)	C12—C17	1.375 (3)
C2—C3	1.399 (2)	C13—C14	1.376 (3)
C3—C4	1.374 (3)	C13—H13	0.9300
C3—H3	0.9300	C14—C15	1.382 (2)
C4—C5	1.368 (3)	C14—H14	0.9300
C4—H4	0.9300	C15—C16	1.372 (2)
C5—C6	1.377 (3)	C16—C17	1.383 (3)
C5—H5	0.9300	C16—H16	0.9300
C6—H6	0.9300	C17—H17	0.9300
S11—O13	1.4462 (12)		
			
C1—N1—H1A	109.5	O12—S11—O11	111.82 (8)
C1—N1—H1B	109.5	O13—S11—C15	106.70 (7)
H1A—N1—H1B	109.5	O12—S11—C15	106.69 (7)
C1—N1—H1C	109.5	O11—S11—C15	106.01 (7)
H1A—N1—H1C	109.5	C12—C11—H11A	109.5
H1B—N1—H1C	109.5	C12—C11—H11B	109.5
C2—N2—H2B	115.8 (19)	H11A—C11—H11B	109.5
C2—N2—H2A	120.5 (15)	C12—C11—H11C	109.5
H2B—N2—H2A	111 (2)	H11A—C11—H11C	109.5
C6—C1—C2	122.28 (14)	H11B—C11—H11C	109.5
C6—C1—N1	118.54 (13)	C13—C12—C17	117.91 (18)
C2—C1—N1	119.16 (12)	C13—C12—C11	121.3 (2)
N2—C2—C1	121.50 (15)	C17—C12—C11	120.8 (2)
N2—C2—C3	121.66 (17)	C12—C13—C14	121.53 (19)
C1—C2—C3	116.73 (16)	C12—C13—H13	119.2
C4—C3—C2	120.76 (18)	C14—C13—H13	119.2
C4—C3—H3	119.6	C13—C14—C15	119.64 (17)
C2—C3—H3	119.6	C13—C14—H14	120.2
C5—C4—C3	121.20 (18)	C15—C14—H14	120.2
C5—C4—H4	119.4	C16—C15—C14	119.92 (16)
C3—C4—H4	119.4	C16—C15—S11	120.67 (13)
C4—C5—C6	119.28 (19)	C14—C15—S11	119.40 (12)
C4—C5—H5	120.4	C15—C16—C17	119.22 (18)
C6—C5—H5	120.4	C15—C16—H16	120.4
C1—C6—C5	119.73 (18)	C17—C16—H16	120.4
C1—C6—H6	120.1	C12—C17—C16	121.77 (18)
C5—C6—H6	120.1	C12—C17—H17	119.1
O13—S11—O12	113.46 (8)	C16—C17—H17	119.1
O13—S11—O11	111.59 (8)		
			
C6—C1—C2—N2	178.01 (16)	C13—C14—C15—C16	0.0 (3)
N1—C1—C2—N2	−0.1 (2)	C13—C14—C15—S11	178.53 (14)
C6—C1—C2—C3	1.7 (2)	O13—S11—C15—C16	−139.99 (14)
N1—C1—C2—C3	−176.40 (13)	O12—S11—C15—C16	−18.38 (16)
N2—C2—C3—C4	−176.72 (18)	O11—S11—C15—C16	100.95 (15)
C1—C2—C3—C4	−0.4 (2)	O13—S11—C15—C14	41.48 (15)
C2—C3—C4—C5	−0.9 (3)	O12—S11—C15—C14	163.08 (13)
C3—C4—C5—C6	0.9 (3)	O11—S11—C15—C14	−77.59 (14)
C2—C1—C6—C5	−1.7 (2)	C14—C15—C16—C17	0.7 (3)
N1—C1—C6—C5	176.40 (16)	S11—C15—C16—C17	−177.78 (15)
C4—C5—C6—C1	0.4 (3)	C13—C12—C17—C16	0.5 (3)
C17—C12—C13—C14	0.3 (3)	C11—C12—C17—C16	−178.0 (2)
C11—C12—C13—C14	178.8 (2)	C15—C16—C17—C12	−1.0 (3)
C12—C13—C14—C15	−0.5 (3)		

### Hydrogen-bond geometry (Å, º)

**Table:**

*D*—H···*A*	*D*—H	H···*A*	*D*···*A*	*D*—H···*A*
N1—H1*A*···O11	0.89	1.89	2.7767 (17)	174
N1—H1*B*···O13^i^	0.89	1.93	2.8111 (17)	171
N1—H1*C*···O12^ii^	0.89	1.92	2.7914 (17)	165
N2—H2*A*···O12	0.90 (2)	2.22 (2)	3.097 (3)	165 (2)

Symmetry codes: (i) −*x*+1, −*y*, −*z*+1; (ii) −*x*+1, −*y*+1, −*z*+1.
